# Should the Pelvic Ring Be Closed After Tumor Resection? A Systematic Review

**DOI:** 10.3390/cancers18111828

**Published:** 2026-06-02

**Authors:** Verena Dammerer, Melanie Ardelt, Johannes Neugebauer, Malena Redl, Markus Neubauer, Gianpaolo Leone, Dietmar Dammerer

**Affiliations:** 1 Department of Orthopedics and Traumatology Hospital Scheibbs, Eisenwurzenstraße 26, 3270 Scheibbs, Austria; 2 Karl Landsteiner University of Health Sciences, Dr. Karl-Dorrek-Straße 30, 3500 Krems, Austria; melanie.ardelt@krems.lknoe.at (M.A.); malena.redl@krems.lknoe.at (M.R.); markus.neubauer@krems.lknoe.at (M.N.); gianpaolo.leone@krems.lknoe.at (G.L.); dietmar.dammerer@krems.lknoe.at (D.D.); 3 Department of Orthopedics and Traumatology, University Hospital Krems, NOE LGA, Karl Landsteiner University, Mitterweg 10, 3500 Krems, Austria; 4 Center for Regenerative Medicine, Department for Health Sciences, Medicine and Research, University for Continuing Education, Dr. Karl-Dorrek-Straße 30, 3500 Krems, Austria

**Keywords:** orthopedic, internal hemipelvectomy, pelvic ring reconstruction

## Abstract

**Background:** Primary malignant bone tumors of the pelvis account for 10–15% of all primary bone sarcomas, most commonly chondrosarcoma, osteosarcoma, and Ewing’s sarcoma. Although advances have shifted treatment toward internal hemipelvectomy, pelvic resections remain challenging due to the complex anatomy. The need for pelvic reconstruction is controversial, balancing potential stability against higher complication rates. This review evaluates the role of pelvic ring reconstruction, focusing on techniques, outcomes, and complications. **Methods:** A systematic literature review was performed in June 2025 using PubMed, MEDLINE and Cochrane Library as the primary databases, with the following search string: (hemipelvectomy) AND (orthopedic), acknowledging that this search strategy may be limited in scope. Studies published within the last five years were considered. After performing a full-text assessment of 80 studies, 14 studies were included in this review. Data regarding patients, methods, and outcomes were extracted and summarized. **Results:** Among the 14 included studies, seven investigated patient-specific three-dimensional (3D) printed pelvic reconstructions, four reported biological reconstruction techniques, two studies focused on non-reconstructive management and one study evaluated alternative stabilization using segmental spinal instrumentation. 3D printed and other reconstructive techniques were associated with improvements in the Musculoskeletal Tumor Society score, reduced pain, and demonstrated osseointegration with few mechanical failures. Although individual case series demonstrated good ambulation and stable fixation, complication rates, particularly wound and infection-related events, remained frequent. Type III reconstructions and personalized implants showed the highest functional gains but occasionally revealed asymptomatic fretting wear. In contrast, the only Level I evidence indicated significantly higher complication and infection rates in reconstructed patients and better functional outcomes in those managed without reconstruction when spinopelvic stability was preserved. Non-reconstructive strategies, including spinal instrumentation, supported early ambulation with low mechanical failure, while pediatric patients treated without reconstruction experienced a high complication rate but acceptable long-term oncologic outcomes. **Conclusions:** Current evidence suggests that routine pelvic ring reconstruction after internal hemipelvectomy may not be justified based on the currently available evidence. Patient-specific 3D-printed implants appear to provide consistent improvements in function, pain reduction, and mechanical stability, but are associated with a relevant risk of wound-related and infectious complications. In patients with preserved spinopelvic stability, non-reconstructive strategies may achieve comparable functional outcomes with lower morbidity. Therefore, pelvic reconstruction should be performed selectively, and further prospective multicenter studies are needed to better define appropriate patient selection and optimize reconstructive strategies.

## Simple Summary

Pelvic bone tumors are rare and difficult to treat because of the complex anatomy and the need for extensive surgery. Removing these tumors can disrupt the pelvic ring, which is important for stability, movement and gait function. Surgeons must decide whether reconstruction of the pelvic ring is necessary after tumor removal. This systematic review summarizes current evidence comparing reconstructive and non-reconstructive approaches. The findings suggest that reconstruction may not always provide additional benefit, especially when pelvic stability is preserved. These insights may support individualized surgical decision-making.

## 1. Introduction

Primary malignant bone tumors are rare, with an overall incidence of approximately 0.9 cases per 100,000 individuals per year in the United States of America [1,2]. About 10–15% of all primary bone sarcomas arise in the pelvic girdle, with osteosarcoma being the most common subtype (35%), followed by chondrosarcoma (30%), and Ewing’s sarcoma (16%) [1,3,4,5]. The pelvis is one of the most common places for both primary and metastatic bone tumors due to its size, hematopoietic tissue content, and complex anatomy [6,7]. Advances in imaging, chemotherapy and surgical techniques have transformed the management of pelvic malignancies, shifting the standard of care from external hemipelvectomy toward limb-salvage procedures such as internal hemipelvectomy [8,9,10]. Despite these developments, pelvic tumor surgery remains technically demanding because of the complex three-dimensional (3D) anatomy and proximity of major neurovascular and visceral structures [9,10]. Nevertheless, it is the cornerstone of curative treatment for most pelvic bone tumors, particularly chondrosarcoma, which is largely resistant to chemotherapy and radiotherapy [5,11].

**Figure 1:**
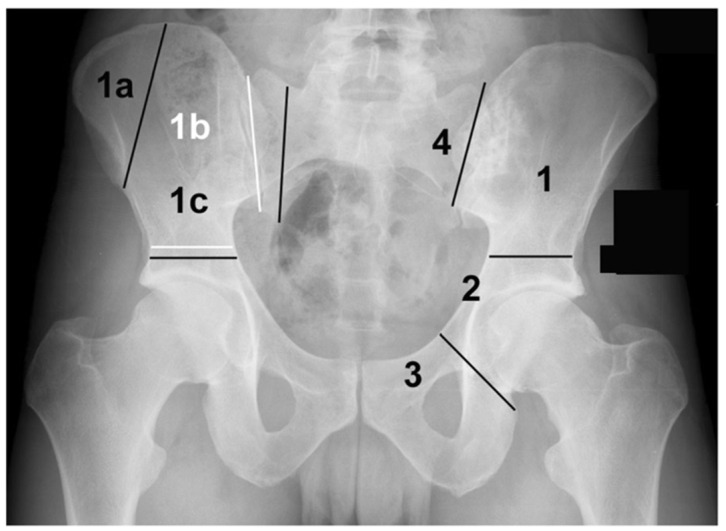
Enneking’s classification of pelvic resections 1 = PI, 2 = PII, 3 = PIII, 4 = PIV [13].

The Enneking and Dunham classification provides a standardized framework for describing pelvic resections (Figure 1) [12,13]. The hemipelvis is divided into four main regions:

- Type I involves the ilium;
- Type II: the periacetabular region;
- Type III: the pubis and ischium;
- Type IV: the sacrum [10,13,14].

Type I resection may be further subdivided into type Ia, partial resections of the iliac wing leaving the pelvic ring intact; type Ib, isolated type I resection; and type Ic, resections including the supraacetabular and the sacroiliac joint, which is a combination of type I and IV [12,13].

Each type of resection is different and presents its own challenges. For example, type II resections need to fix restoration of hip function and joint stability using either endoprosthetic or biological reconstruction; while, in contrast, type I and type III resections mainly disrupt pelvic ring continuity but generally preserve hip articulation [10,15]. In the past, type III defects were left unreconstructed, as the principal weight-bearing axis remained intact. However, there is growing evidence that losing the anterior pelvic ring can change how the pelvis moves, increase the forces on the sacroiliac joint, and lead to long-term complications such as pelvic instability, osteoarthritis, and hernia formation [16,17,18].

The Musculoskeletal Tumor Society (MSTS) score is a standardized system used to evaluate functional outcomes after limb-salvage (Table 1) [19]. It assesses six key categories, including pain, function, emotional acceptance, and three limb-specific abilities, each rated from zero to five points [19,20]. The individual ratings are combined into a total numerical or percentage score, allowing reliable comparison of functional results across different studies and surgical techniques [19].

Despite significant progress, there is still a lot of discussion about whether and how to reconstruct the pelvic ring after tumor resection. Some authors argue that reconstruction may not improve function significantly and instead increases the risk of complications, while others emphasize its biomechanical and structural importance for maintaining pelvic stability, reducing pain, and preventing deformity [9,17,18].

Considering these conflicting views, a clear evaluation of the necessity and impact of pelvic ring closure after tumor resection is still lacking. Therefore, the aim of this study is to assess whether pelvic ring reconstruction provides a measurable clinical and biomechanical benefit following tumor resection.

## 2. Materials and Methods

**Figure 2:**
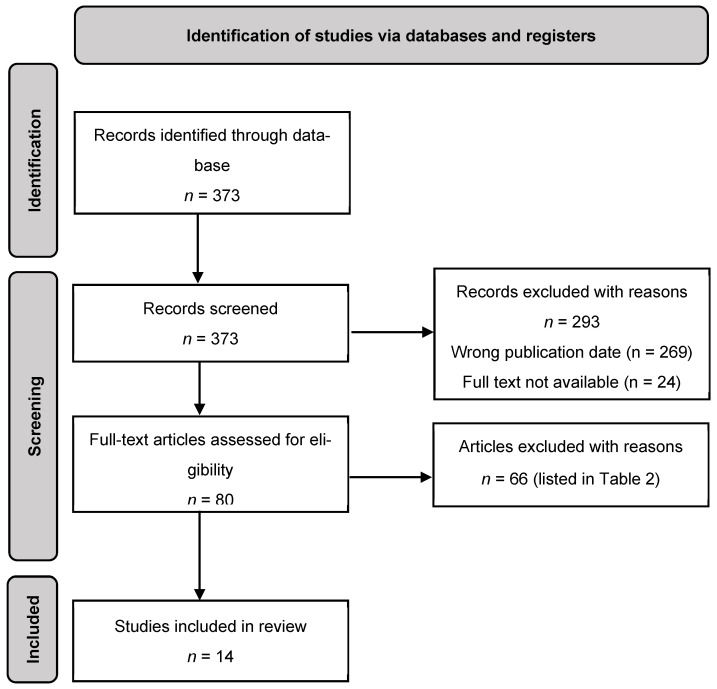
PRISMA flowchart of literature research and analysis.

A comprehensive literature search was conducted by two independent researchers in June 2025 using PubMed, MEDLINE, and the Cochrane Library. Disagreements were discussed and handled according to the listed inclusion and exclusion criteria. A systematic review was performed in accordance with the Preferred Reporting Items for Systematic Reviews and Meta-Analyses (PRISMA, Supplementary Materials) guidelines, compare Figure 2 [21]. The protocol for the systematic review of our study was not registered for the International Prospective Register of Systematic Reviews (PROSPERO). The following search terms were used to screen literature: (hemipelvectomy) AND (orthopedic) on 5 June 2025. After applying the filters “publication date: 5 years” and “full text available”, a total of 80 studies were identified. Following a full-text assessment of the remaining papers, 14 studies were included in the final analysis. A detailed description of the exclusion criteria is provided in Table 2, and the main characteristics and outcomes of the included studies are summarized in Table 3.

## 3. Results

Overall, 14 studies including approximately 400 patients undergoing pelvic tumor resection with or without reconstruction were analyzed. Study designs consisted of one systematic review (Level I), six retrospective cohort studies (Level III), three retrospective case series and four case reports (Level IV). Mean patient age ranged from 13.4 to 65.3 years, with pediatric cohorts representing a minority and most adult studies reporting mean ages between 33 and 53 years. Reported follow-up periods ranged from 6 to 110 months, with the majority of studies providing mid- to long-term follow-up of approximately 24 to 48 months. Across studies, outcomes were predominantly assessed using the MSTS score, VAS or NRS pain scales, ambulation status, radiographic implant integration, and postoperative complication rates, particularly wound healing disorders and infections. The included studies investigated a broad spectrum of surgical approaches, which can be categorized into 3D-printed personalized reconstruction, biological or hybrid reconstruction techniques, and resection without reconstruction or alternative stabilization techniques. Overall, the included studies were considered to be at moderate to high risk of bias, primarily due to retrospective designs, small sample sizes, and inconsistent outcome reporting.

Seven of the 14 studies analyzed 3D-printed, patient-specific implants for pelvic reconstruction [3,4,6,14,15,16,17]. The studies evaluating 3D-printed implants generally reported functional improvement, with MSTS scores increasing from preoperative values of 12–15 to postoperative values ranging between 20 and 30 points, corresponding to a good to excellent functional outcome [3,4,6,14,15,16,17]. Pain scores demonstrated a consistent reduction, with VAS or NRS values decreasing from around 5 preoperatively to 1–2 at final follow-up (mean 38 months) [3,4,15,17]. Radiographic evaluation suggested high rates of successful osseointegration, with minimal interfacial gaps and stable bone-implant interfaces [3,4,16,17]. Mechanical complications appeared to be infrequent, with low rates of implant failure, aseptic loosening, or fracture, and implant survival rates of over 70% [3,4,6,14,15,16,17]. Overall complication rates ranged from 14 to 17%, with wound healing disorders and infections representing the most frequent adverse events [3,4]. Hip dislocation, screw-related complications, or implant-specific structural problems occurred sporadically and were generally manageable without long-term functional limitations [4,6,15]. Across different defect types, including acetabular, pubic, and combined type I-III resections, most patients retained or regained ambulatory ability, frequently without assistive devices [6,16,17]. Biomechanical analyses indicated stress concentration mainly at the sacral fixation points and the lumbosacral junction, without translating into clinically relevant implant failure during follow-up [6].

Biological and hybrid reconstruction approaches, including iliac crest grafting, femur upshifting with total hip arthroplasty, and hip transposition, resulted in moderate-to-good functional outcomes, with reported MSTS scores ranging from 19 to 28 points [5,11,22,23]. However, these techniques were associated with higher soft-tissue complication rates, particularly wound necrosis and deep infection [22]. Early postoperative recovery and ambulation appeared to correlate positively with functional outcomes, although a substantial proportion of patients required additional surgical procedures for wound management [22].

Resection without reconstruction was primarily reported in pediatric cohorts and in the systematic review [8,9]. In pediatric patients, complication rates reached up to 64.7%, including nerve injury, infection, and flap necrosis, while gait outcomes ranged from normal to abnormal despite acceptable oncologic control [8]. The systematic review demonstrated that reconstruction was associated with significantly higher overall complication rates compared with no reconstruction (62.3% vs. 25.7%) and higher infection rates (13.7% vs. 0%) [9]. Functional outcomes favored non-reconstructed patients, with MSTS scores of 82% compared with 71.6% in reconstructed cohorts, particularly in the absence of spinopelvic instability [9].

Alternative stabilization techniques, such as segmental spinal instrumentation, enabled very early ambulation (median 5 days), provided durable mechanical stability, and showed no hardware loosening or failure, despite occasional wound-related complications [10].

Overall, 3D-printed personalized reconstructions appeared to demonstrate the most consistent balance between functional recovery, pain reduction, and mechanical reliability, at the cost of moderate wound-related complication rates. Biological and hybrid reconstructions achieved acceptable functional recovery but were associated with higher soft-tissue morbidity and reintervention rates. Non-reconstruction strategies were associated with lower complication rates and potentially favorable functional outcomes, particularly when spinopelvic stability was preserved.

## 4. Discussion

This review evaluated 14 studies published over the past five years to analyze whether reconstruction of the pelvic ring after internal hemipelvectomy is indicated and shows advantages. The included evidence demonstrates that modern reconstruction techniques, particularly patient-specific 3D-printed implants, suggest encouraging functional outcomes, reliable osseointegration, and low rates of mechanical failure [3,4,16,17]. Across multiple cohorts, MSTS score improvements appeared to be relevant, pain consistently decreased, ambulation was preserved, and radiological integration appeared robust even in complex defects involving multiple pelvic regions [3,4,6,16,17]. Case-based and small-cohort reconstructions also showed good restoration of stability, pain-free gait, and no recurrence in the short to mid-term period [14,23]. However, despite these functional advantages, reconstruction was frequently associated with a considerable complication burden, predominantly wound-related problems and infections [3,4,9,22].

In this context, it is important to note that there is still no clear consensus on key aspects of periprosthetic joint infection (PJI) management in orthopedic oncology [24]. A recent expert consensus found strong agreement on infection prophylaxis, early aggressive management of persistent wound drainage, and surgical revision strategies, while short-duration antibiotic prophylaxis was considered sufficient in lower-risk reconstructions [24]. Single-stage, 1.5-stage, and two-stage revisions were all regarded as valid options, with two-stage revision remaining the most reliable approach [24]. In contrast, no consensus was reached on the role of debridement, antibiotics, and implant retention (DAIR) or prolonged antibiotic therapy after revision [24]. Given that wound complications and infections are the most frequent adverse events after pelvic reconstruction, these findings support a cautious and selective indication for pelvic ring reconstruction, balancing biomechanical benefits against infection-related morbidity [24].

The decision to reconstruct the pelvic ring following tumor resection remains one of the most debated issues in musculoskeletal oncology. Traditionally, reconstruction of the anterior pelvic ring was often considered ineffective, particularly after type III hemipelvectomy, as the weight-bearing axis through the acetabulum and sacrum was considered to be preserved [11]. However, recent evidence suggests that disruption of the anterior ring can result in altered pelvic biomechanics, leading to increased shear stress on the sacroiliac joint, pelvic instability, pain and hernia formation [11,16,18]. Cases of bladder herniation following unreconstructed type III resections have also been reported, prompting several authors to recommend anterior reconstruction in order to restore pelvic integrity and prevent soft tissue complications [11,18]. Various reconstructive methods have been described following pelvic tumor resection, including autografts, allografts, myocutaneous flaps, prosthetic mesh, and modular or custom-made hemipelvic prostheses [3,4,15,17]. While biological reconstructions offer biocompatibility, they are predisposed to infection, graft resorption and mechanical failure, whereas endoprosthetic reconstructions provide early stability and functional recovery but may suffer from aseptic loosening or infection [6,15].

Recently, 3D printing has enabled the production of patient-specific implants that anatomically restore pelvic continuity and promote osseointegration through porous titanium structures [4,6,17]. These findings are consistent with multiple studies included in this review, in which large patient cohorts demonstrated reliable osseointegration and accurate implant fit following 3D-printed hemipelvic reconstruction [4,16,17]. Modern reconstructive techniques, particularly 3D-printed customized prostheses, have demonstrated promising early outcomes by enabling precise anatomical matching, improved osseointegration and mechanical reliability [3,4,16,17,18]. Those in favor of reconstruction emphasize that maintaining a continuous pelvic ring ensures mechanical stability, reduces asymmetric loading and provides a structural base of soft tissue reattachment [4,6,16,17,18]. Restoring ring continuity has been associated with improved pain relief, enhanced gait function and a lower risk of sacroiliac stress concentration [4,6,18]. Integrating porous titanium structures promotes bone ingrowth and enhances the bone-prosthesis interface, contributing to stable, long-lasting results and improved limb function [3,4,16,17]. Furthermore, reconstruction enables the reattachment of abdominal and adductor muscles, thereby reinforcing the pelvic floor and preventing postoperative hernias [6,16,18]. Studies have reported high MSTS scores (up to 28–30 points), satisfactory early function and marked pain reduction in patients following complete pelvic ring reconstruction using custom-made 3D-printed implants [4,16,17]. Nevertheless, even with advanced implant design, complications remain common. Wound healing disorders, infections, and implant-specific mechanical issues were reported [3,4,9]. Biomechanical concerns, such as stress concentration at sacral fixation points, fretting wear around prosthetic stems, and impaired physiological micromotion of the pelvic ring, highlight that even advanced implants may not perfectly reproduce physiological loading patterns [6,18]. Although 3D-printed reconstructions demonstrate improved short-term outcomes, long-term data are limited and the optimal balance between rigidity and physiological motion remains to be determined [4,6,18].

Biological and hybrid reconstruction approaches achieved moderate-to-good functional outcomes but were associated with higher soft tissue morbidity and reintervention rates [11,22,23]. Several case reports highlight that individualized reconstruction strategies, such as femur upshifting or combined 3D-printed prosthetic and arthrodesis techniques, can restore stable, pain-free function even in complex defect situations [14,23]. Importantly, the only Level I evidence identified in this review demonstrated that reconstruction was associated with significantly higher overall complication and infection rates compared with no reconstruction, while functional outcomes were superior in patients who did not undergo reconstruction when spinopelvic stability was preserved [9]. These findings indicate that reconstruction is not universally beneficial and may not be indicated in all cases with intact posterior pelvic support or limited biomechanical disruption [9]. In pediatric patients, individualized decision-making appears particularly important, as small anatomical structures and fragile tissues increase the risk of iatrogenic injury during complex reconstruction procedures [8]. Junjua et al. [8] demonstrated that pediatric patients treated without reconstruction experienced high complication rates but achieved long-term disease-free survival in most cases.

In summary, this review suggests that reconstruction of the pelvic ring after internal hemipelvectomy should not be considered a routine procedure. Given the substantial global disparities in access to specialized sarcoma care and advanced reconstruction techniques, the generalizability of complex pelvic reconstruction strategies remains limited and further supports a selective, indication-based approach [25]. While 3D-printed patient-specific implants provide the most consistent balance between functional recovery, pain reduction, and mechanical reliability, they remain associated with a moderate but relevant complication burden. Non-reconstructive strategies demonstrated early mobilization and fewer complications when spinopelvic stability is preserved. Therefore, reconstruction should be reserved for selected cases with clear mechanical indication, such as loss of posterior pelvic stability, large bone defects, or significant sacroiliac joint involvement, emphasizing the importance of individualized, defect-specific treatment planning [9,18].

Internal hemipelvectomy with pelvic ring reconstruction using patient-specific implants is associated with considerable costs, as custom 3D-printed prostheses typically range from USD 20,000 to 40,000 and may require up to two months for manufacturing [26]. Some reports estimate the design and production cost of a customized hemipelvic implant at approximately USD 15,000 [27]. However, the overall treatment costs are influenced more by postoperative complications and longer hospital stays than by the implant itself [28]. Compared with non-reconstructive surgery, reconstruction with implants has been associated with a 5.7-fold increase in total costs, although only about 10% of this increase is directly related to the implant [28].

## 5. Complications

Despite the potential biomechanical and functional benefits, pelvic ring reconstruction is associated with a high overall complication rate. Reported complication rates following pelvic reconstruction for any resection type range from 50 to 60%, substantially increasing the likelihood of secondary surgery and long-term morbidity [9]. Extensive reconstructions are associated with prolonged operative time, increased blood loss, a higher risk of infection, wound complications, neurological deficits and mechanical failure, particularly when metallic implants or grafts are used [8,9,10,16]. In extreme cases, persistent deep infection has necessitated prosthesis removal or even secondary external hemipelvectomy [10].

Newer 3D-printed patient-specific implants have demonstrated improved complication profiles compared with historical reconstruction techniques [29]. Infection rates were reduced to approximately 15% and no dislocations, loosening, or structural failures were reported at a mean follow-up of 27 months [15]. Nevertheless, wound infections and healing disorders are the most frequent complications [3,4,29]. Biomechanical considerations remain relevant in this group. Rigid anterior fixation may disrupt the physiological micromotion of the pubic symphysis, resulting in stress concentration and implant-related complications [6,18]. Biomechanical studies suggest that a semi-rigid or flexible connection that mimics the natural fibrous symphysis may reduce fretting wear or sacroiliac irritation [6,18]. Clinical findings from our review support this concept, as radiographic fretting wear was observed in patients with type III 3D-printed prostheses, although notably without associated symptoms [17].

Autograft and allograft reconstructions have shown particularly high reoperation rates, while massive allografts are prone to fracture or resorption [10]. Soft-tissue complications, including wound necrosis and deep infection, were frequently reported in biological reconstructions [22].

In contrast, resection without reconstruction was associated with lower overall complication and infection rates, particularly in the absence of spinopelvic instability [9,10]. A recent systematic review showed that patients undergoing reconstruction after sacroiliac or hemipelvis resection experienced higher complication rates and slightly poorer functional outcomes compared to non-reconstructed patients [9].

Historical custom-made implants were associated with poor outcomes, including dislocation rates of up to 20%, aseptic loosening rates of up to 24%, structural complication rates of up to 33%, and infection rates exceeding 50% [10,15]. Similarly, non-custom modular endoprostheses continue to demonstrate high complication rates, with reported dislocation rates ranging from 4 to 26%, aseptic loosening from 0 to 16%, and infection rates from 14 to 47% [15].

Postoperative pain constitutes a relevant early complication following hemipelvectomy and may require prolonged multimodal analgesic management to enable functional recovery [25]. A pediatric series demonstrated that pain intensity peaked on postoperative day zero, day five, and day 30, with all patients requiring opioid-based analgesia in combination with acetaminophen and adjunctive agents such as regional anesthesia, gabapentin, ketamine, or non-steroidal anti-inflammatory drugs [30]. Although pain scores generally declined throughout the postoperative course and acceptable pain control was achieved, acute postoperative pain necessitated continuous evaluation to facilitate early mobilization and rehabilitation [30].

Impairment of gait and walking capacity is a frequent postoperative consequence of hemipelvectomy due to loss of hip muscles, altered pelvic biomechanics, and changes in neuromuscular control [31,32]. Instrumented gait analyses and patient-specific neuromusculoskeletal modeling have demonstrated reduced self-selected walking speed, increased stance width, compensatory trunk lean, and significant alterations in muscle coordination despite only minor visual gait abnormalities [31]. Clinical series report delayed recovery of ambulation, with approximately 70–76% of patients achieving walking with bilateral crutches within two months postoperatively, reflecting the prolonged rehabilitation required after pelvic resection [22]. Computational simulations further suggest that strengthening of key muscles such as the psoas may influence postoperative walking ability and energy efficiency, highlighting the biomechanical vulnerability of gait after hemipelvectomy [32].

Postoperative scoliosis is a common long-term complication after hemipelvectomy, with approximately 30% of patients developing a Cobb angle of over 10°, often with rapid progression during the first postoperative year [33]. Identified risk factors for scoliosis development include external hemipelvectomy, extensive pelvic resections, iliac crest involvement, resection of L5/sacrum, and the absence of pelvic ring reconstruction [33]. Scoliosis progression may result in back pain, radiculopathy, functional worsening, and impaired walking ability, occasionally necessitating secondary spinal surgery [33,34]. Case reports and biomechanical analyses indicate that pelvic deformity and spinopelvic instability after tumor resection can contribute to persistent or progressive spinal deformity [34,35]. Accurate pelvic reconstruction and careful postoperative spinal monitoring are therefore considered essential to minimize the risk of structural scoliosis and its long-term functional consequences [33,35].

These findings highlight the technical complexity and long learning curve associated with pelvic tumor reconstruction [29]. Preventive strategies aimed at reducing complication rates include careful surgical technique, systemic optimization, repeated intraoperative irrigation with saline and povidone-iodine, and the use of broad-spectrum antibiotics [16].

## 6. Limitations

Although a broad search string was applied to maximize sensitivity, it may have resulted in the inclusion of highly heterogeneous studies in design, patient populations, reconstruction techniques, and outcome reporting, which limits comparability and reduces the strength of the conclusion. Many reports involved very small sample sizes or single cases, thereby reducing the reliability and generalizability of functional and complication outcomes. Follow-up durations were often short, and radiological or biomechanical assessments were inconsistently documented, restricting insight into long-term implant performance. Future research should focus on larger multicenter cohorts with standardized reporting and long-term follow-up to better evaluate functional outcomes, complications, and implant longevity, to significantly compare and clarify the balance between functional benefit and complication risk of reconstruction versus non-reconstruction of the pelvic ring.

## 7. Conclusions

Taken together, the available evidence suggests that pelvic ring reconstruction should not be performed routinely after internal hemipelvectomy, but should be reserved for selected patients with clear biomechanical instability, large anterior defects, or relevant soft-tissue deficiency. While modern reconstruction techniques, particularly patient-specific 3D-printed implants, may improve pain, function, and mechanical stability, they remain associated with a relevant risk of wound-related and infectious complications. In contrast, non-reconstructive strategies often achieve comparable or superior functional outcomes with fewer complications when spinopelvic integrity is preserved. Therefore, reconstruction should follow an individualized, indication-based approach rather than a routine strategy. Future studies should focus on prospective, multicenter designs with standardized outcomes and longer follow-up to better define patients who truly benefit from reconstruction and to further optimize implant design and fixation strategies.

## Figures and Tables

**Table 1:** MSTS score for lower limb [19].

Parameter	0 Points (0%)	1 Point (3.3%)	2 Points (6.7%)	3 Points (10%)	4 Points (13.3%)	5 Points (16.7%)
Pain	Severe	Marked	Moderate	Mild	Occasional	None
Function	Non-functional	Very limited	Limited	Moderate	Near normal	Normal
Emotional acceptance	Poor	Fair	Moderate	Good	Very good	Excellent
Supports	Full	Heavy	Moderate	Minimal	Occasional	None
Walking	Unable	Very limited	Short distances	Moderate distances	Long distances	Normal
Gait	Non-functional	Severe limp	Moderate limp	Slight limp	Nearly normal	Normal

**Table 2:** Description of the exclusion criteria.

Exclusion Criteria (*n* = 66)
- (23) Surgical technique description without patient data - (14) Studies focusing exclusively on postoperative complications (e.g., pain, scoliosis, gait impairment, fractures; not eligible for analysis addressing pelvic ring closure) - (10) Traumatology-related studies without neoplastic disease - (6) Studies from other medical specialties (e.g., urology, general surgery, radiology) - (4) Quality of life, patient experience, or survival studies (no analysis of pelvic ring reconstruction) - (4) Rare case reports (exceptional cases without generalizable conclusions) - (2) Incorrect tumor localization (Femoral tumors) - (2) Neoplasia overview articles - (1) Animal study

**Table 3:** Characteristics of the studies involved in the current study.

Author (Year, Country)	Patient Number (Time)	Age (Mean)	Methods	Follow-Up (Months)	Outcomes	Level of Evidence *	Study Design
Patient-specific 3D-printed pelvic reconstructions							
Hu (2025, CHN) [3]	12 (2018–2023)	37.3	3D-printed custom hemipelvic reconstruction using auxetic porous implants	48 m	– MSTS improved 15→26 – VAS pain 5→1 – Complications: 16.7% delayed wound healing – No mechanical failure, loosening, or fracture – Complete osseointegration	III	Retrospective
Hu (2024, CHN) [4]	96 (2017–2022)	47.4	3D-printed hemipelvic reconstruction	48.1 m	– MSTS improved 12.2→23.8 – VAS pain 5.3→1.4 – Complications: 13.5% (6.3% wound healing, 4.2% infection, 2.1% dislocation, 1% screw fracture/loosening) – Precise implantation and complete bone-prosthesis integration	III	Retrospective
Guo (2023, CHN) [6]	4 (2019–2022)	53	Reconstruction with 3D-printed prostheses after type I + II + III internal hemipelvectomy	16.6 m	– MSTS: 19.5 – Preserved ambulation – Complications: 1 hip dislocation, no implant-related complication – favorable biomechanical characteristics	IV	Retrospective
Khal (2023, ROU) [14]	1	25	Internal hemipelvectomy and reconstruction with custom-made 3D printed prosthesis, hip arthroplasty and ilio-sacral arthrodesis	24 m	– MSTS: 28 – Excellent function – Good osteointegration – No complications or recurrence	IV	Case report
Broekhuis (2023, NLD) [15]	15 (2013–2018)	33.9	3D printed titanium pelvic implant to reconstruct the hip joint after internal hemipelvectomy	33.8 m	– MSTS: 63.3% (19p.) – NRS pain: rest 0, activity 2 – Implant-related complications in 4 patients: implant survival 73.3% – No aseptic loosening	IV	Retrospective
He (2025, CHN) [16]	4 (2017–2021)	42	3D-printed bilateral pubis prosthesis reconstruction	15.5 m	– MSTS: 28.5 – Ambulation without devices in all patients – Complications: 1 incision necrosis – No implant failure or displacement – Good osteointegration in all cases	IV	Retrospective
Zhang (2021, CHN) [17]	5 (2017–2019)	36.6	Type III hemipelvectomy and 3D-printed customized prosthesis reconstruction	23.6 m	– MSTS: 29.8 – VAS pain 5→1 – Complications: 1 erectile dysfunction, 3 fretting wear without symptoms – No deep infection, dislocation, or recurrence	IV	Retrospective
Biological reconstruction techniques							
Escudero-Acurio (2022, CHL) [5]	1	64	Type I-II internal hemipelvectomy and total hip prosthesis	10 m	– Ambulation with assistance, painless gait – Excellent hip motion – Complication: postoperative deep infection – No recurrence or implant loosening	IV	Case report
Murphy (2021, AUS) [11]	1 (2019)	54	Anterior hemipelvectomy and reconstruction with iliac crest graft	6 m	– Pain-free independent ambulation – Complication: urinary tract infection – No recurrence	IV	Case report
Zhang (2023, JPN) [22]	38 (2009–2018)	46	Internal hemipelvectomy and reconstruction with hip transposition	17 m (6–110 m)	– MSTS early vs. delayed recovery: 17 vs. 14 – Ambulation: 76% walking with bilateral crutches at 60 days – Complications: 42% required additional surgery for wound problems (flap necrosis, infection)	III	Retrospective
Mahran (2023, EGY) [23]	1 (2019)	16	Pelvic Ewing’s sarcoma: Femur upshifting and total hip prosthesis	36 m	– MSTS: 76.7% – Independent ambulation – Stable implant position – No recurrence	IV	Case report
Non-reconstructive management							
Junjua (2025, PAK) [8]	17 (2010–2018)	13.4	Internal hemipelvectomy in pediatric patients without reconstruction	34.4 m (8–96 m)	– Ambulation: 58.8% normal gait, 41.2% abnormal gait – Complications: 64.7% (nerve injury, infection, flap necrosis, recurrence) – Disease-free survival: 70.6% – Mortality: 29.4%	IV	Retrospective
Zavras (2022, USA) [9]	201	33.9	Internal hemipelvectomy with type IV or I/IV resection, comparison of reconstructive vs. non-reconstructive	52.3 m (2–240 m)	– MSTS higher without reconstruction (82% vs. 71.6%) – Overall complication rate: 54.8% (14.4% wound complications) – Reconstruction associated with higher complications (62.3% vs. 25.7%) and infections (13.7% vs. 0%) – Non-reconstruction may yield superior outcomes in patients without spinopelvic junction instability	I	Systematic review
Alternative reconstruction with segmental spinal instrumentation							
Tepper (2020, USA) [10]	4 (2015–2020)	65.3	Pelvic ring reconstruction after type I or I/IV resection with segmental spinal instrumentation	48 m	– Rapid ambulation (median 5 days) – Complications: 2 wound necrosis, 1 infection; no neurologic or visceral injury – No hardware failure or loosening – Oncologic outcomes: 1 death (metastases), 1 recurrence	IV	Retrospective

* Evidence levels were assigned according to commonly used classification systems; due to small sample sizes and retrospective designs, several studies were classified as Level IV.

## Data Availability

No new data were created or analyzed in this study. The original contributions presented in this study are included in the article. Further inquiries can be directed to the corresponding author.

## Supplementary Materials

The following supporting information can be downloaded at: https://www.mdpi.com/article/10.3390/cancers18111828/s1, PRISMA 2020 Checklist.
